# Characteristics of NH_4_^+^ and NO_3_^−^ Fluxes in *Taxodium* Roots under Different Nitrogen Treatments

**DOI:** 10.3390/plants11070894

**Published:** 2022-03-28

**Authors:** Shuting Wu, Jianfeng Hua, Yan Lu, Rui Zhang, Yunlong Yin

**Affiliations:** Jiangsu Engineering Research Center for Taxodium Rich, Germplasm Innovation and Propagation, Institute of Botany, Jiangsu Province and Chinese Academy of Sciences, Nanjing Botanical Garden, Memorial Sun Yat-Sen, Nanjing 210014, China; salvia_wu@163.com (S.W.); 18260093866@163.com (Y.L.); zr202022@outlook.com (R.Z.); ylyin@cnbg.net (Y.Y.)

**Keywords:** N management, net fluxes, productivity, proton, non-invasive micro-test technique

## Abstract

To understand the characteristics of net NH_4_^+^ and NO_3_^−^ fluxes and their relation with net H^+^ fluxes in *Taxodium*, net fluxes of NH_4_^+^, NO_3_^−^ and H^+^ were detected by a scanning ion-selective electrode technique under different forms of fixed nitrogen (N) and experimental conditions. The results showed that higher net NH_4_^+^ and NO_3_^−^ fluxes occurred at 2.1–3.0 mm from the root apex in *T.* *ascendens* and *T. distichum*. Compared to NH_4_^+^ or NO_3_^−^ alone, more stable net NH_4_^+^ and NO_3_^−^ fluxes were found under NH_4_NO_3_ supply conditions, of which net NH_4_^+^ flux was promoted at least 1.71 times by NO_3_^−^, whereas net NO_3_^−^ flux was reduced more than 81.66% by NH_4_^+^ in all plants, which indicated that NH_4_^+^ is preferred by *Taxodium* plants. *T. ascendens* and *T. mucronatum* had the largest net NH_4_^+^ and total N influxes when NH_4_^+^:NO_3_^−^ was 3:1. ^15^N Atom% and activities of N assimilation enzymes were improved by single N fertilization in the roots of *T. distichum*. In most cases, net H^+^ fluxes were tightly correlated with net NH_4_^+^ and NO_3_^−^ fluxes. Thus, both N forms and proportions could affect N uptake of *Taxodium*. These findings could provide useful guidance for N management for better productivity of *Taxodium* plants.

## 1. Introduction

Nitrogen (N) plays a significant role in plant growth and development since it is a crucial component of plants’ chlorophylls, nucleic acids, proteins, and secondary metabolites [1]. Ammonium (NH_4_^+^) and nitrate (NO_3_^−^) are two primary forms of inorganic N absorbed and used by plants, and their fluxes in roots are varied with the distance from the apex. Spatial variability in the fluxes of NH_4_^+^ and NO_3_^−^ has been explored along the roots in some herbaceous and woody plants [2,3,4,5]. For instance, the maximal net NH_4_^+^ influx happened at the root apex in rice (*Oryza sativa* L.) [2] and *Populus simonii* [5], whereas the highest net NH_4_^+^ influx appeared at 5 mm, 10 mm, and 5–20 mm from the root apex in lodgepole pine (*Pinus contorta*) [6], *Populus popularis* [7] and Douglas-fir (*Pseudotsuga menziesii*) [6], respectively. In the case of NO_3_^−^, previous studies observed that the highest net NO_3_^−^ flux occurred at 0–10 mm in *P. contorta* [6], and at 15 mm from the apex in *P. simonii* [5] and *P. popularis* [7]. In rice, net NO_3_^−^ influx increased to a maximum at 21 mm from the apex and then gradually declined [2]. Obviously, different plant species have distinct patterns of NH_4_^+^ and NO_3_^−^ flux rates along the fine roots.

Apart from the spatial variation along the roots, NH_4_^+^ and NO_3_^−^ fluxes are also affected by environmental factors such as N levels. A previous study in tea (*Camellia sinensis*) roots demonstrated increased net influxes of NH_4_^+^ and NO_3_^−^ when the solution concentration increased from 0.2 mM to 1.2 mM under KNO_3_ and NH_4_Cl [1]. However, research on *Picea glauca* revealed a converse result in most cases; the roots presented net NH_4_^+^ and NO_3_^−^ influxes in 50 μM with net effluxes in 1500 μM solutions [8]. When roots were treated in 10, 100, and 1000 μM NH_4_NO_3_ solutions, net NH_4_^+^ influxes increased gradually in *P. popularis* but decreased by degrees in *Populus alba* × *Populus glandulosa* [4]. In contrast, the hybrid presented higher net NO_3_^−^ influxes than *P. popularis* in most cases [4]. This phenomenon revealed that the N concentrations in soils have prominent effects on the uptake of NH_4_^+^ and NO_3_^−^, and they are significantly related to the plant species. Additionally, previous studies revealed that interactions between NH_4_^+^ and NO_3_^−^ exist on fluxes of both ions [7,9]. The interactions between NH_4_^+^ and NO_3_^−^ are complicated among plants [10], and the underlying mechanisms remain unclear [7]. It is documented that the presence of NH_4_^+^ and NO_3_^−^ negatively affect the uptake of each other, but NH_4_^+^ is preferred in *C. sinensis* [1]. However, net NH_4_^+^ influx was induced by the simultaneous provision of NO_3_^−^, and net NO_3_^−^ influx was inhibited in the presence of NH_4_^+^ in roots of *P. popularis* and *Populus asperata* [7,11]. Moreover, a previous study on Douglas-fir and lodgepole pine showed that net NH_4_^+^ uptake remained unchanged in the presence or absence of NO_3_^−^ [6]. Overall, interactions between NH_4_^+^ and NO_3_^−^ and their preferences may result in changes of NH_4_^+^ and NO_3_^−^ fluxes under different proportions of NH_4_^+^ and NO_3_^−^ supply. Nonetheless, little information is available on the fluxes of NH_4_^+^ and NO_3_^−^ in plant roots under fluctuating proportions of both inorganic N forms.

On the other hand, fluxes of NH_4_^+^ and NO_3_^−^ are correlated with the plasma membrane PM-H^+^-ATPase activity that extrudes H^+^ from the cytosol to the outside at the expense of adenosine triphosphate (ATP) [12]. Previous research found that NO_3_^−^ is transported across the plasma membrane via NO_3_^−^/H^+^ symporters with the involvement of PM-H^+^-ATPase [13]. The concentration of NH_4_^+^ can increase the activity of PM-H^+^-ATPase [14]. Furthermore, the expression of genes encoding PM-H^+^-ATPase was positively associated with fluxes of NH_4_^+^ and NO_3_^−^ [8]. The significant correlations between NH_4_^+^, NO_3_^−^ fluxes, and H^+^ uptake rate have been observed in many plants [12,15,16,17].

*Taxodium* species including *T*. *ascendens*, *T. distichum*, and *T. mucronatum* have been introduced from southeastern America to many countries owing to their economic and ecological benefits [18]. For instance, they can be used as woody bioenergy crops [19]. *Taxodium* oil showed adequate bioassay for insecticidal activity [20]. Compounds isolated from the bark can exhibit cytotoxic substances, thus treating against cancer cells [21]. Moreover, *Taxodium* plants have been selected as suitable species for afforestation in many challenging areas [22,23]. Although N is crucial for *Taxodium* growth and development, less information is available on the fluxes of NH_4_^+^ and NO_3_^−^ as well as their correlation with H^+^ flux in fine roots. In this study, a non-invasive micro-electrodes technique was employed to investigate NH_4_^+^, NO_3_^−^ and H^+^ fluxes in fine roots of *T. ascendens, T. distichum*, and *T. mucronatum* under different N forms and their proportions. Our objectives were (i) to determine the distance from the root apex of *Taxodium* plants where there are greater net NH_4_^+^ and NO_3_^−^ fluxes; (ii) to illustrate the characteristics of NH_4_^+^, NO_3_^−^ and H^+^ fluxes and their interactions under different N forms and proportions.

## 2. Materials and Methods

### 2.1. Plant Cultivation

Semi-lignified cuttings (10 cm in length, 0.3 cm in diameter) of *T. ascendens*, *T. distichum*, and *T. mucronatum* were selected. After being soaked in 3‰ 3-indoleacetic acid (IAA) solution for 2 min, they were repotted into a pot containing 1:1 volume of peat: perlite in a ventilated greenhouse at the Institute of Botany, Jiangsu Province and Chinese Academy of Sciences (35° 03′ N, 118° 49′ E), under normal growth conditions (approximately 25 °C) in a photoperiod of 14/10 h of light/dark. Two months later, cuttings with uniform size and development were selected for the NMT experiments.

Seeds were collected from a healthy *T. distichum* grown in the Institute of Botany, Jiangsu Province and Chinese Academy of Sciences. They were planted in black plastic pots (5 × 5 × 15 cm) filled with 1:1 volume of peat:perlite in a climate chamber (23–25 °C/15–18 °C, day/night; light per day, 14 h; photosynthetic photon flux, 160 μmol m^−2^ s^−1^; relative air humidity, 50–60%). After 19 weeks of growth, the plants were transferred into black plastic boxes (25 × 15 × 14.5 cm, 4 plants per box) containing 4 L of modified 1/4 Hoagland’s nutrient solution [24]. All nutrient solutions were continuously aerated with an air pump, and each solution was refreshed every other day. After 16 d, plants were used to explore ^15^N Atom% and enzymatic activities.

### 2.2. Experimental Design

To determine the positions along the root where the maximal influxes of NH_4_^+^ and NO_3_^−^ occur, a preliminary experiment was carried out at 14 positions, in turns, 0, 0.3, 0.6, 0.9, 1.2, 1.5, 1.8, 2.1, 2.5, 3.0, 5.0, 8.0, 15.0 and 30.0 mm away from the root apex. The measuring solution was 0.5 mM MES (2-(N-Morpholino) ethanesulfonic acid hydrate buffer.), pH 6.0, to which either 1.0 mM NH_4_Cl for NH_4_^+^ or 1.0 mM KNO_3_ for NO_3_^−^ was added. After that, the position where the greater net uptake of NH_4_^+^ and NO_3_^−^ occurred was detected to carry out the following experiments.

To investigate the net fluxes of NH_4_^+^ and NO_3_^−^ under different N forms and proportions, the measuring solutions were designed as (1) NH_4_^+^ (NH_4_Cl): 0.1 and 1.0 mM, (2) NO_3_^−^ (KNO_3_): 0.1 and 1.0 mM and (3) NH_4_^+^:NO_3_^−^: 1:3, 1:1, and 3:1 (total N = 2 mM), containing 0.5 mM MES, pH 6.0.

To explore the biomass, ^15^N Atom% and enzymatic activities under single N fertilization, 24 seedlings with similar performance (ca. 15 cm in height) were selected and divided into three groups (8 plants in each group). Three N treatments: 0 mM ^15^NH_4_Cl and K^15^NO_3_ (serving as control, CK), 1 mM ^15^NH_4_Cl and 1 mM K^15^NO_3_ in 1/4 modified Hoagland’s nutrient solution [24] were applied. Dicyandiamide (7 μM, C_2_H_4_N_4_) was added into the nutrient solution to inhibit nitrification [19]. After 3 d, 4 plants from each treatment were harvested and used for measurements of ^15^N Atom%, and the remaining 4 plants in each treatment were used for enzymatic activities.

### 2.3. Measurement of NH_4_^+^, NO_3_^−^ and H^+^ Fluxes

**Figure 1:**
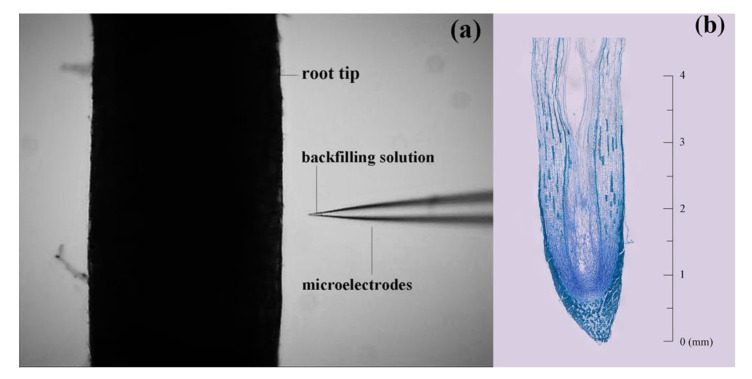
Root tip in NMT experiment (**a**) and the vertical section in the root of *T. distichum* (**b**).

To understand the real-time NH_4_^+^, NO_3_^−^ and H^+^ uptake by the fine roots under different treatments, ions flux alterations on the root surface were measured by using a non-invasive micro-test technology (NMT) system (youngerusa.com; xuyue.net) (Figure 1a).

The measurement procedures were described by Zhao et al. [5]. Firstly, ion-selective microelectrodes designed with 2–4 μm apertures were manufactured and silanized. Secondly, for the NH_4_^+^ electrode, in sequence, 100 mM NH_4_Cl was used as a backfilling solution, followed by an NH_4_^+^ selective liquid ion exchange cocktail (#09879, Sigma, St. Louis, MI, USA). Similarly, for the NO_3_^−^ electrode, 10 mM KNO_3_ was used as the backfilling solution, followed by a NO_3_^−^ selective liquid ion exchange cocktail (#72549, Sigma). For the H^+^ electrode, 15 mM NaCl and 40 mM KH_2_PO_4_ were used as backfilling solution, followed by an H^+^ selective liquid ion exchange cocktail (#95293, Sigma). Prior to the flux measurements, the microelectrodes were calibrated. For NH_4_^+^ calibration, 0.05/0.5 mM NH_4_Cl in addition to other compounds (0.5 mM MES, pH 6.4/5.4) were used in the measuring solution; for NO_3_^−^ calibration, 0.05/0.5 mM KNO_3_ in addition to the compounds (0.5 mM MES, pH 6.4/5.4) were used in the measuring solution; for H^+^ calibration, pH 6.4/5.4 in addition to 0.5 mM MES were used in the measuring solution. Only electrodes with Nernstian slopes higher than 55 mV per tenfold concentration difference were used.

After that, fine white roots, 15–35 mm from the apex, were selected. They were fixed at the bottom of the petri dish filled with 10–20 mL measuring solutions for 20 min. After being equilibrated, the samples were transferred to another petri dish containing 5 mL fresh solution and then were put under the microscope. The tips of the microelectrodes were aligned and kept 30 μm away from the target point, which is a specific distance from the apex of the root. Net fluxes of NH_4_^+^/NO_3_^−^ were recorded at each measurement point for 5 min. Not only eight biological repetitions (eight fine roots from four plants) but also 50 measurement time points in each repetition were considered.

### 2.4. Determination of ^15^N Uptake and Enzyme Activities

The roots were harvested and rinsed three times in distilled water. The ^15^N Atom% (^15^N AT%) and the amount of plant N derived from ^15^N-labeled fertilizer (*Ndff*%) were detected [25].

Activities of nitrate reductase (NR, EC 1.7.99.4), nitrite reductase (NiR, EC 1.7.2.1), glutamine synthetase (GS, EC 6.3.1.2), glutamate synthetase (GOGAT, EC 1.4.7.1) and glutamate dehydrogenase (GDH, EC 1.4.1.2) in the roots were assayed [4].

### 2.5. Statistical Analysis

In order to determine the NH_4_^+^, NO_3_^−^ and H^+^ fluxes along the root tip, imFluxes V2.0 (xuyue.net) was used to obtain the data at each measuring point. The positive values represent net influxes, and the net negative values represent net effluxes. To analyze data for the ion fluxes, biomass, ^15^N AT% and enzyme activities, one-way ANOVA (Duncan’s multiple range tests at 5% level) was performed with the SPSS 25.0 (Statistical Product and Service Solutions, IBM, New York, NY, USA). GraphPad Prism version 9.1 was used to draw figures.

## 3. Results

### 3.1. Net Fluxes of NH_4_^+^ and NO_3_^−^ along the Root Tip

**Figure 2:**
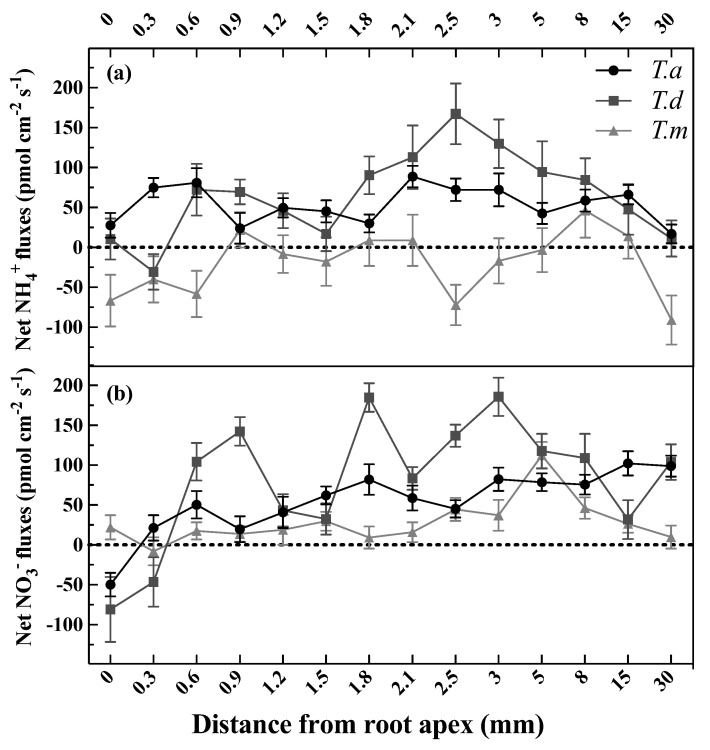
Net NH_4_^+^ (**a**) and NO_3_^−^ (**b**) fluxes along the root tip of *T. ascendens*, *T. distichum*, and *T. mucronatum*. Bars indicate standard errors (*n* = 8). Net influxes correspond to positive values, and negative values indicate net effluxes, respectively. The concentrations of N were set as 1.0 mM NH_4_Cl for NH_4_^+^ or 1.0 mM KNO_3_ for NO_3_^−^. *T. a*, *T. d*, and *T. m* represent *T. ascendens*, *T. distichum* and *T. mucronatum*, respectively.

Net fluxes of NH_4_^+^ and NO_3_^−^ were determined along the root tip up to 30.0 mm from the apex, and their fluxes were widely varied at different locations (Figure 2a,b). The NH_4_^+^ fluxes ranged from 17.02 (net influx) to 88.89 (net influx) pmol cm^−2^ s^−1^ in *T. ascendens*, and varied dramatically from −30.52 to 167.15 pmol cm^−2^ s^−1^ in *T. distichum*, and fluctuated between −90.94 and 46.31 pmol cm^−2^ s^−1^ in *T. mucronatum* when supplied as 1 mM NH_4_Cl (Figure 2a). Intriguingly, both *T. ascendens* and *T. distichum* showed strong NH_4_^+^ uptake rates from 2.1 to 3.0 mm along the root tip (Figure 2a).

Net NO_3_^−^ fluxes ranged from −49.68 to 102.15 pmol cm^−2^ s^−1^ in *T. ascendens*, from −80.61 to 185.98 pmol cm^−2^ s^−1^ in *T. distichum*, and from −7.88 to 112.27 pmol cm^−2^ s^−1^ in *T. mucronatum* when fed with 1 mM KNO_3_ (Figure 2b). The maximal net NO_3_^−^ influxes of *T. ascendens*, *T. distichum*, and *T. mucronatum* were detected at 15.0, 3.0, and 5.0 mm from the root apex, respectively (Figure 2b). As a result, the following experiments selected 2.5 mm from the root apex as the specific position to investigate the net fluxes of NH_4_^+^ and NO_3_^−^. Moreover, 2.5 mm from the apex belongs to the elongation zone of the root tip in *Taxodium* plants (Figure 1b).

### 3.2. Net Fluxes of NH_4_^+^ and NO_3_^−^ under Different N Forms

**Figure 3:**
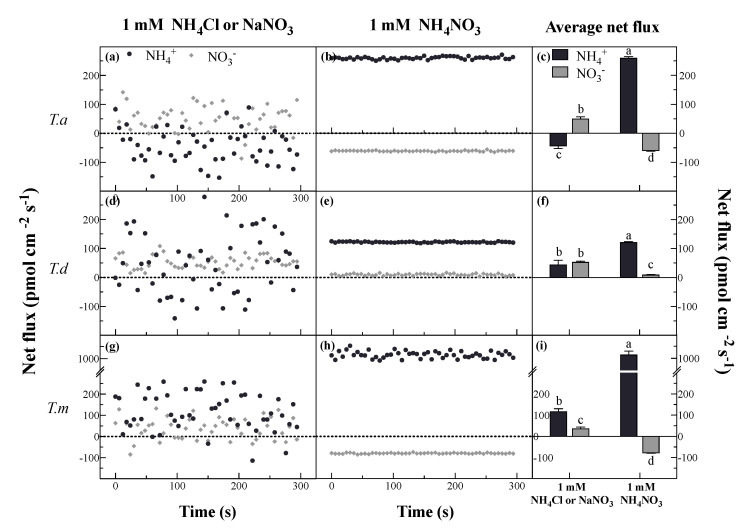
Net fluxes of NH_4_^+^ and NO_3_^−^ under single (**a**,**d**,**g**) and mixed (**b**,**e**,**h**) N forms, and the means of net fluxes of NH_4_^+^ and NO_3_^−^ (**c**,**f**,**i**). Bars indicate standard errors (*n* = 8). Different letters indicate significant differences among the treatments according to Duncan’s Multiple Range Test at 5% level. *T. a*, *T. d*, and *T. m* represent *T. ascendens*, *T. distichum* and *T. mucronatum*, respectively.

As NH_4_Cl and KNO_3_ were added separately, the NH_4_^+^ and NO_3_^−^ fluxes fluctuated widely for all tested plants at 2.5 mm from the root apex during a 5-min period (Figure 3). Both NH_4_^+^ and NO_3_^−^ fluxes of *T. distichum* and *T. mucronatum* showed a tendency towards net influx. *T. ascendens*, however, tended to show net efflux of NH_4_^+^ and net influx of NO_3_^−^ (Figure 3a,d,g). When supplied with mixed N (NH_4_NO_3_), stable fluxes of NH_4_^+^ and NO_3_^−^ were observed, and distinctly, NH_4_^+^ fluxes were much greater than NO_3_^−^ fluxes in all *Taxodium* plants (Figure 3b,e,h). Compared to 1 mM NH_4_Cl, average net fluxes of NH_4_^+^ were stimulated by 688%, 171%, and 762% under 1 mM NH_4_NO_3_ in roots of *T. ascendens*, *T. distichum,* and *T. mucronatum*, respectively (Figure 3c,f,i). Thus, the increase of NH_4_^+^ fluxes was as follows: *T. mucronatum > T. ascendens* > *T. distichum* (Figure 3c,f,i). The same order was observed for the decreases in net NO_3_^−^ fluxes, which were decreased by 314%, 220%, and 81.66% under 1 mM NH_4_NO_3_ compared with that under 1 mM KNO_3_, respectively (Figure 3c,f,i).

### 3.3. Net NH_4_^+^, NO_3_^−^ and H^+^ Fluxes under Different N Concentrations

**Figure 4:**
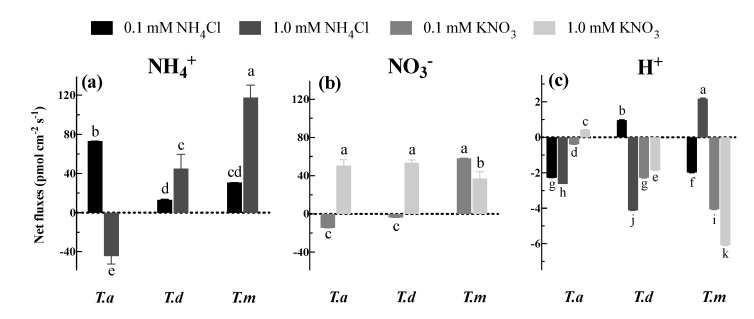
Net fluxes of NH_4_^+^ (**a**), NO_3_^−^ (**b**) and H^+^ (**c**) under different concentrations of NH_4_Cl and KNO_3_. Bars indicate standard errors (*n* = 8). Different letters indicate significant differences among the treatments according to Duncan’s Multiple Range Test at 5% level. *T. a*, *T. d*, and *T. m* represent *T. ascendens*, *T. distichum* and *T. mucronatum*, respectively.

Except for *T. ascendens* exposed to 1.0 mM NH_4_Cl, all three species showed a tendency for net NH_4_^+^ influx when fed with 0.1 or 1.0 mM NH_4_Cl (Figure 4a). Additionally, the net influx of NH_4_^+^ in *T. ascendens* was significantly greater (*p* < 0.05) than those in the other two species under 0.1 mM NH_4_Cl (Figure 4a). Compared to 0.1 mM NH_4_Cl, net influx of NH_4_^+^ was promoted by 2.40 and 2.84 times under 1.0 mM NH_4_Cl treatment in *T. distichum* and *T. mucronatum*, respectively (Figure 4a). Apart from *T. ascendens* and *T. distichum* treated with 0.1 mM KNO_3_, all the plants displayed a tendency for net NO_3_^−^ influx when supplied with 0.1 or 1.0 mM KNO_3_ (Figure 4b). Moreover, the fluxes of NO_3_^−^ were significantly lower (*p* < 0.05) in 1.0 mM than in 0.1 mM KNO_3_ in *T. mucronatum* (Figure 4b).

At the same time, net H^+^ fluxes were determined in this study (Figure 4c). Here, we found that H^+^ presented net effluxes under all treatments except for *T. ascendens* under 1.0 mM KNO_3_, *T. distichum* under 0.1 mM NH_4_Cl, and *T. mucronatum* under 1.0 mM NH_4_Cl (Figure 4c). Other than *T. distichum* exposed to 0.1 mM NH_4_Cl, the change tendency of net H^+^ fluxes was similar to the variations of net NH_4_^+^ and NO_3_^−^ fluxes when the solution concentration was increased from 0.1 mM to 1.0 mM (Figure 4c).

### 3.4. Net NH_4_^+^ and NO_3_^−^ Fluxes under Different N Proportions

**Figure 5:**
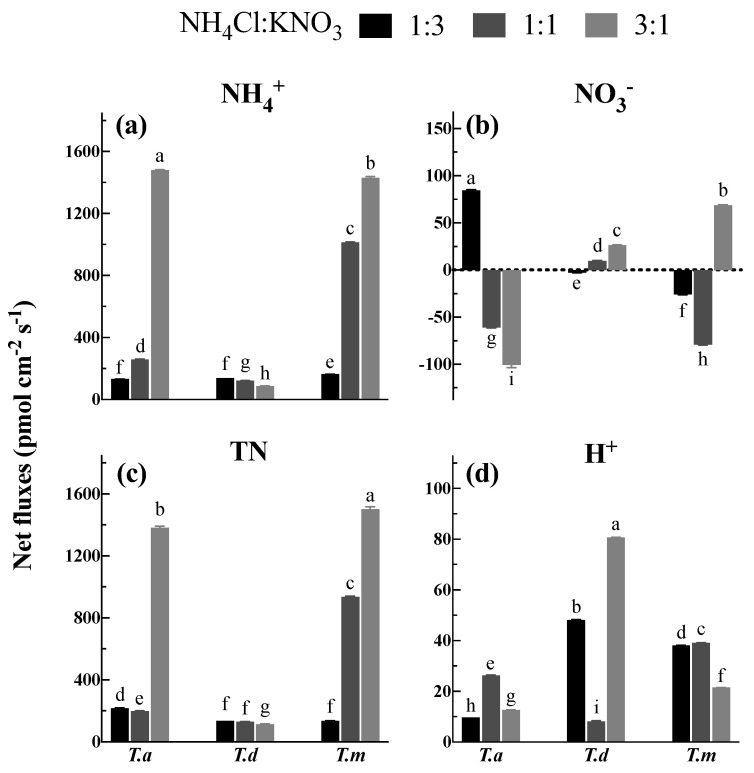
Net fluxes of NH_4_^+^ (**a**), NO_3_^−^ (**b**), total N (**c**) and H^+^ (**d**) under different proportions of NH_4_Cl and KNO_3_. Bars indicate standard errors (*n* = 8). Different letters indicate significant differences among the treatments according to Duncan’s Multiple Range Test at 5% level. *T. a*, *T. d*, and *T. m* represent *T. ascendens*, *T. distichum* and *T. mucronatum*, respectively.

Under 2.0 mM TN (total nitrogen) consisting of various proportions of NH_4_Cl and KNO_3_ (1:3, 1:1, and 3:1), *T. ascendens*, *T*. *distichum,* and *T. mucronatum* showed great diversities in the fluxes of NH_4_^+^, NO_3_^−^ and TN (Figure 5). The net influx of NH_4_^+^ ranged from 88.00 to 1480.80 pmol cm^−2^ s^−1^ across the three tested species (Figure 5a). It was 0.96 and 10.15 times greater under NH_4_: NO_3_ at 1:1 and 3:1 than at 1:3 in *T. ascendens*, respectively (Figure 5a). Similarly, the net NH_4_^+^ influxes were 5.21 and 7.76 times higher than 1:3 when treated with 1:1 and 3:1, respectively, in *T. mucronatum* (Figure 5a). A decreasing trend of net NH_4_^+^ influx was observed for *T*. *distichum* with an increase in the NH_4_^+^ proportion (Figure 5a).

Compared with the net NH_4_^+^ influx, the net flux of NO_3_^−^ was much lower, ranging from −100.53 to 84.85 pmol cm^−2^ s^−1^ under different proportions of NH_4_Cl and KNO_3_ (Figure 5b). It is surprising that the net influx of NO_3_^−^ observed under the 1:3 solution was replaced by net efflux when the proportion changed to 1:1 and 3:1 in *T. ascendens* (Figure 5b). *T. distichum*, however, presented a totally converse trend whereby the net flux of NO_3_^−^ significantly (*p* < 0.05) increased by 1.68 times when the NH_4_^+^ proportion was raised from 1:1 to 3:1 (Figure 5b). In the case of *T. mucronatum*, there was net efflux under 1:3 and 1:1 and net influx under 3:1 (Figure 5b).

The trend of TN fluxes ranging from 114.23 to 1500.48 pmol cm^−2^ s^−1^ was similar to the fluxes of NH_4_^+^ (Figure 5c), and the highest net influx of TN was observed for *T. mucronatum* and *T. ascendens* when the proportion of NH_4_Cl: KNO_3_ was 3:1 (Figure 5c). Among the three species, *T. distichum* displayed the lowest net NH_4_^+^, NO_3_^−^ and TN fluxes in all measuring solutions (Figure 5c).

In addition, the net H^+^ fluxes were determined in this study (Figure 5d). All the treatments showed net H^+^ influx when the two forms of N were provided together (Figure 5d).

### 3.5. ^15^N AT%, Ndff% and Enzyme Activities in the Roots of T. distichum

Compared with CK, ^15^N AT% was elevated by 70.27% or 29.73% in 1 mM ^15^NH_4_^+^-treated or 1 mM ^15^NO_3_^−^-treated *T.distichum* roots (Table 1). Similar results were also observed in *Ndff%* (Table 1). Compared to CK, however, no significant difference was found in the root biomass of *T. distichum* supplied with 1 mM ^15^NH_4_^+^ or ^15^NO_3_^−^ during the 3 d experiment period (Table 1).

^15^NH_4_^+^ or ^15^NO_3_^−^ fertilization also has positive impacts on the activities of N assimilation enzymes (Table 2). When compared with CK, NR, NiR, GS, GDH and GOGAT activities were enhanced by 50.63%, 33.74%, 39.40%, 48.25% and 59.36%, respectively in 1 mM ^15^NH_4_^+^-supplied *T. distichum* roots (Table 2). Similarly, the activities of NR and NiR were increased by 221.03% and 11.93%, respectively in 1 mM ^15^NO_3_^−^-fed *T. distichum* roots (Table 2).

## 4. Discussion

### 4.1. Spatial Variability of Net NH_4_^+^ and NO_3_^−^ Fluxes along the Fine Roots

Fine roots consist of four distinct regions, including root cap, meristematic, elongation, and maturation zones, characterized by different anatomical and functional features [7]. These anatomical and functional diversities could bring about distinct absorbing abilities for NH_4_^+^ and NO_3_^−^ in different root zones [26,27,28]. Spatial variability of net NH_4_^+^ and/or NO_3_^−^ flux has been observed in fine roots of various plant species [29]. For example, maximal net NH_4_^+^ influx occurred at the root apex in rice [2] and *P. simonii* [5], and at 5 mm, 10 mm, and 5–20 mm from the root apex in *P. contorta* [6] and *P. popularis* [7], and Douglas-fir [6] respectively. Such spatial variation of net NH_4_^+^ and NO_3_^−^ influxes along the root axis was also observed in our research. The largest net influxes of NH_4_^+^ and NO_3_^−^ were detected at m from the apex of *T*. *ascendens* and *T. distichum,* which belongs to the elongation zone. Such differences are possibly because of cytosolic concentrations of NH_4_^+^ and NO_3_^−^ in the elongation zone being lower than the thresholds needed for N assimilation to support the fast growth [30,31]. Similar results were observed in studies of *Arabidopsis*, where larger net NH_4_^+^ fluxes were shown in the elongation zones [28,32]. Moreover, *Phyllostachys edulis* showed relatively higher net influxes of NH_4_^+^ and NO_3_^−^ at 2–5 mm from the root apex [30]. The net NH_4_^+^ or NO_3_^−^ fluxes were found to be higher in segment I (0–35 mm) than segment II (35–70 mm) in *Populus* × *canescens* [31]. In addition, we found that *T. distichum* had the greatest NH_4_^+^ and NO_3_^−^ uptake rates among the three *Taxodium* species.

### 4.2. Net NH_4_^+^ and NO_3_^−^ Fluxes under Single N Treatments

Generally, environmental N levels have a significant impact on the NH_4_^+^ and NO_3_^−^ fluxes of fine roots [4]. For instance, gradual increases in the fluxes of NH_4_^+^ and/or NO_3_^−^ were determined when supplied N was elevated in *P. popularis* and *P. alba* × *P. glandulosa* [4], and *C. sinensis* [1]. However, the opposite results were observed in *P. glauca* [8], wheat [24], and corn (*Zea mays* L.) [33]. In our study, except for *T. ascendens* under NH_4_Cl and *T. mucronatum* under KNO_3_ solutions, most outcomes showed elevated NH_4_^+^ or NO_3_^−^ uptake rates resulting from increasing NH_4_Cl or KNO_3_ supply. Consistently, higher ^15^N AT% and *Ndff%* were induced by ^15^NH_4_^+^ or ^15^NO_3_^−^ treatment in the roots of *T. distichum*. Moreover, ^15^NH_4_^+^ or ^15^NO_3_^−^ fertilization also brought about higher activities of NR, NiR, GS, GDH and GOGAT in the roots of *T. distichum*. These results suggest that N fertilization could be applied to stimulate NH_4_^+^ and NO_3_^−^ absorption and assimilation capacities for *Taxodium* plants in practice.

Although an increasing N supply is likely to enhance N uptake in most cases, the provision of just NH_4_^+^ could lead to soil acidification [34]. In most cases, to maintain ion homeostasis, roots release H^+^ while absorbing NH_4_^+^, decreasing pH in the growth medium [35,36]. Eventually, this may lead to physiological and morphological disturbance of plants and then bring about toxicity and low production [37]. For example, acidification can significantly induce aluminum absorption, which is harmful to the development of plants [38]. In contrast, after absorption of NO_3_^−^, OH^−^ could be released, contributing to the increase of pH [39]. Thus, a balanced supply of NH_4_^+^ and NO_3_^−^ is expected to improve the N uptake of plants and the soil environment.

### 4.3. Net NH_4_^+^ and NO_3_^−^ Fluxes under Mixed N Treatments

Many studies had demonstrated that the uptake of NH_4_^+^ and NO_3_^−^ was affected by each other when both N forms were provided [1,7]. In this study, the presence of NO_3_^−^ stimulated the uptake of NH_4_^+^, whereas the net fluxes of NO_3_^−^ were inhibited by NH_4_^+^ in *Taxodium* plants. Similar results were found in the roots of corn, tea, wheat, rice and *Brassica campestris* [1,2,9,24,33], which indicated that NH_4_^+^ and NO_3_^−^ might interact with each other under coexistence N forms. These results might be related to cytosolic NH_4_^+^/NO_3_^−^ thresholds [30]. In detail, more NH_4_^+^ may be required for plant development when NO_3_^−^ was provided, while pre-existing NH_4_^+^ may reduce the thresholds of NO_3_^−^ in the plant [1]. Considering that a higher net NH_4_^+^ influx than NO_3_^−^ was observed, it can be concluded that *Taxodium* plants show a preference for NH_4_^+^. It is noted that when NH_4_Cl or KNO_3_ was solely supplied, the fluxes of NH_4_^+^ or NO_3_^−^ in the three species were erratic. However, stable net NH_4_^+^ and NO_3_^−^ fluxes were observed when NH_4_^+^ and NO_3_^−^ were both present in the solution, indicating a better balance in the mixed solution. In addition, this interesting phenomenon was reported in the study of *C. sinensi*, which might be the result of the competition between NH_4_^+^ and NO_3_^−^, and the underlying mechanism needs to be further studied [1].

Because of the greater N uptake in mixed treatments than in single N conditions, strong net uptake of NH_4_^+^ in fine roots of *Taxodium* species was expected to occur when NH_4_^+^ and NO_3_^−^ were supplied in different proportions [37]. In the case of tea, the maximum net NH_4_^+^ influx was observed when NH_4_^+^:NO_3_^−^ was 1:1, and the highest net NO_3_^−^ influx occurred when NH_4_^+^:NO_3_^−^ was 1.2:1 [1]. In blueberry (*Vaccinium corymbosum* L.), the mRNA levels of *ammonium transporter 3* (*VcAMT3*) involved in NH_4_^+^ uptake as well as *nitrate transporter 1.5* (*VcNRT1.5*) and *VcNRT2* involved in NO_3_^−^ uptake was highest when the NH_4_^+^:NO_3_^−^ ratio was 2:1 [40]. The highest growth rate, which is positively correlated with N uptake, of *T. aestivum* L., *Brachiaria brizantha,* and *Pseudostellaria heterophylla* was found when the NH_4_^+^ and NO_3_^−^ were supplied equivalently [1,24,41,42]. In this study, the best uptake rates of N were found when NH_4_^+^:NO_3_^−^ was 3:1, 1:3, and 3:1 for *T. ascendens*, *T. distichum,* and *T. mucronatum,* respectively, which could provide an applicable proportion of NH_4_^+^ and NO_3_^−^ when producing special N fertilizer for the productivity of *Taxodium* plants. Additionally, the optimal equilibrium between NH_4_^+^ and NO_3_^−^ supply largely differed between the three *Taxodium* species, implying that the induction of N transport systems require distinct NH_4_^+^ and NO_3_^−^ ratios among these plants.

In the present study, we found that with the change in the proportion of NH_4_^+^: NO_3_^−^ (total N concentration: 2 mM), the NH_4_^+^ influxes were improved more than NO_3_^−^. This observation indicates a preference for NH_4_^+^ over NO_3_^−^, which is in good agreement with our previous outcomes. In most plant species, NH_4_^+^ is first absorbed into cells and then directly converted to amino acids, whereas cytosolic NO_3_^−^ is assimilated at a higher energy cost. It is reduced to NO_2_^−^ with the help of nitrate reductase (NR) and is further converted into NH_4_^+^ in plastids by nitrite reductase (NiR), which requires more energy than NH_4_^+^ for both transportation and further reduction [43]. On the other hand, the flux discrepancies between NH_4_^+^ and NO_3_^−^ might result from the lower activity of NO_3_^−^ transport systems affected by NH_4_^+^, which reduces the expression of the NO_3_^−^-related genes [1]. In blueberry plants, the expression of AMTs and NRTs was largely affected by the different ratios of NH_4_^+^: NO_3_^−^ [40]. A previous study has indicated that different AMTs determined the uptake of NH_4_^+^ to a certain extent, which was mediated by the external concentration [44]. Furthermore, various AMTs and NRTs have different substrate affinities appropriate to different N concentrations [5,8,10]. Therefore, the complicated fluxes of NH_4_^+^ and NO_3_^−^ when supplied at different proportions might be related to the distinct energetic and biochemical characteristics of uptake and assimilation pathway between NH_4_^+^ and NO_3_^−^ in plant roots [4,5,24].

### 4.4. Net NH_4_^+^ and NO_3_^−^ Fluxes Associated with H^+^

In this study, the alteration of H^+^ fluxes was tightly associated with the variation in NH_4_^+^ or NO_3_^−^. Previous studies revealed that H^+^ fluxes might be correlated with the transport of NH_4_^+^ and NO_3_^−^, since NH_4_^+^ is transported into root cells through a symporter (co-transport with H^+^) and/or a uniporter, and NO_3_^−^ is co-transported with H^+^ via a symporter into the cytosol [12,13,14]. Additionally, by maintaining a proton gradient, plasma membrane PM-H^+^-ATPase facilitates transport by pumping H^+^ into the apoplast during the uptake of NH_4_^+^ or NO_3_^−^ in some parts of the roots [4,43,45]. The activities of PM-H^+^-ATPase are determined by the transcript levels of corresponding mRNAs [4]. Although inconsistent results of H^+^ fluxes under different N treatments were observed in the present study, H^+^ still plays an essential role in plant uptake of NH_4_^+^ and NO_3_^−^. Similar to a previous study in fine roots of *P. popularis* [7], our data indicated a tendency for net H^+^ uptake when two forms of N were supplied simultaneously. Intriguingly, fluxes of H^+^ fluctuated under both single N sources. Through our findings, we suspect that there may be an interaction between net H^+^ flux and net NH_4_^+^/NO_3_^−^ flux in roots of *Taxodium* species. Similar results were observed by Garnett et al. [15]. The specifics of the proposed interaction remain unclear. It is challenging to find out the specific mechanism underlying the correlation between H^+^ and NH_4_^+^/NO_3_^−^ in *Taxodium* roots.

## 5. Conclusions

In summary, spatial variability of NH_4_^+^ and NO_3_^−^ fluxes was observed along fine roots of *Taxodium* plants, and *T. ascendens* and *T. distichum* had higher fluxes of NH_4_^+^ and NO_3_^−^ at 2.1–3.0 mm from the root apex. In most cases, net fluxes of NH_4_^+^ and NO_3_^−^ increased with the elevated single N levels. NH_4_^+^ and NO_3_^−^ affected each other when they were both supplied, and *Taxodium* plants preferred NH_4_^+^. Higher net N influxes were found when NH_4_^+^ and NO_3_^−^ were simultaneously supplied than sole N treatments, especially in *T. ascendens* and *T. mucronatum* at 3:1 of NH_4_^+^:NO_3_^−^. Additionally, H^+^ fluxes were tightly correlated with net NH_4_^+^ and NO_3_^−^ fluxes. These findings are valuable for understanding the characteristics of NH_4_^+^ and NO_3_^−^ fluxes in the fine roots of *Taxodium* plants in the context of single and various ratios of N supply, and could provide a scientific basis for N management for silvicultural practice and better productivity of *Taxodium* plants.

## Figures and Tables

**Table 1:** Biomass, ^15^N AT% and *Ndff%* in the roots of *T. distichum* under three N treatments.

Treatments	Root Biomass/g	^15^N AT%	*Ndff*%
CK	0.30 ± 0.01 ^bc^	0.37 ± 0.00 ^c^	/
1 mM ^15^NH_4_^+^	0.34 ± 0.02 ^ab^	0.63 ± 0.03 ^a^	2.65 ± 0.31 ^a^
1 mM ^15^NO_3_^−^	0.26 ± 0.01 ^c^	0.48 ± 0.04 ^b^	1.16 ± 0.38 ^b^

Different letters behind the values in the same column indicate significant differences among the treatments according to Duncan’s Multiple Range Test at 5% level. ^15^N AT%: ^15^N Atom; *Ndff*%:^15^N from N source.

**Table 2:** Activities of N assimilation enzymes in the roots of *T. distichum* under three N treatments.

Treatments	NR Activity μmol h^−1^ mg^−1^ Protein	NiR Activity μmol h^−1^ mg^−1^ Protein	GS Activity μmol h^−1^ mg^−1^ Protein	GDH Activity μmol h^−1^ mg^−1^ Protein	GOGAT Activity μmol h^−1^ mg^−1^ Protein
CK	0.58 ± 0.02 ^c^	2.43 ± 0.04 ^c^	0.33 ± 0.00 ^b^	1.04 ± 0.06 ^b^	0.78 ± 0.03 ^b^
1 mM ^15^NH_4_^+^	0.87 ± 0.02 ^b^	3.25 ± 0.08 ^a^	0.46 ± 0.22 ^a^	1.54 ± 0.08 ^a^	1.24 ± 0.03 ^a^
1 mM ^15^NO_3_^−^	1.92 ± 0.04 ^a^	2.72 ± 0.01 ^b^	0.34 ± 0.01 ^b^	1.01 ± 0.04 ^b^	0.74 ± 0.01 ^b^

Different letters behind the values in the same column indicate significant differences between the treatments according to Duncan’s Multiple Range Test at 5% level. NR: nitrate reductase; NiR: nitrite reductase; GS: glutamine synthetase; GDH: glutamate dehydrogenase; GOGAT: glutamate synthetase.

## Data Availability

Not applicable.
